# Disability-Specific Associations with Child Health and Functioning

**DOI:** 10.3390/ijerph16061024

**Published:** 2019-03-20

**Authors:** Ko Ling Chan, Camilla K. M. Lo, Frederick K. Ho, Patrick Ip

**Affiliations:** 1 Department of Applied Social Sciences, The Hong Kong Polytechnic University, Hong Kong, China; 2 Department of Paediatrics and Adolescent Medicine, The University of Hong Kong, Hong Kong, China; fredkho@connect.hku.hk (F.K.H.); patricip@hku.hk (P.I.)

**Keywords:** child physical disabilities, learning and developmental disabilities, intellectual disabilities, internalizing disorders, autism spectrum disorder, health-related quality of life, child health

## Abstract

This study examined the health profile of children with different types of disabilities and explored the disability-specific associations with various types of health and functioning using a large nonclinical sample of children. A cross-sectional school survey was conducted during 2016 and 2017. A total of 4114 children (aged 6–18 years) receiving primary or secondary education, or their proxy, in Hong Kong participated in the study. Disabilities were categorized as (a) physical disabilities; (b) learning and developmental disabilities; (c) intellectual disabilities; (d) internalizing disorders or mental illness; and (e) autism spectrum disorder. Health-related quality of life (QoL), sleep-related QoL, activities of daily living (ADL), emotional functioning, and social functioning were assessed and compared between children with disabilities and those without. The results showed that children with disabilities showed poorer physical functioning, health-related QoL, and emotional and social functioning than their counterparts without disabilities. Disability-specific associations with health were found: (a) physical disabilities and intellectual disabilities were associated with greater difficulties in ADL; (b) language impairment and Attention deficit/ hyperactivity disorder (ADHD) were negatively associated with sleep-related QoL; (c) all types of disabilities but hearing impairment were negatively associated with health-related QoL (HRQoL); and (d) language impairment, ADHD, internalizing disorder, as well as autism spectrum disorder were associated with greater abnormal behavioral difficulties. The findings warrant the development of tailor-made intervention programs and give insights to effective resource allocation for the children in need.

## 1. Introduction

According to the World Health Organization (WHO), disabilities or impairments affect approximately 5% of children worldwide [1]. Disability can be conceptualized on “a continuum from minor difficulties in functioning to major impacts on a person’s life” (p.22) [2]. Children with disabilities can encompass children who exhibit a variety of physical, sensory, cognitive, developmental, learning, intellectual, emotional, and behavioral disorders [3,4].

As a vulnerable population, children with disabilities have consistently been found with greater risk of hampered health and functioning. For example, findings from empirical and meta-analytic studies have revealed significantly poorer mental health or increased risk of mental problems among children with physical disabilities [5], intellectual disabilities [6], autistic spectrum disorders [7], and learning disabilities [8]. Furthermore, other research has shown hampered social skills and functioning among children with disabilities such as physical disabilities [9], autistic spectrum disorder [10], and learning disabilities [11].

Reliable estimates of the scope of the issue are crucial for the development of effective intervention programs for children with different types of disabilities in order to improve their health and health-related quality of life (HRQoL) [12]. However, most existing studies either used small clinical samples when investigating the associations between disabilities and health, or focused on only one type of disability or one aspect of health in their investigation, thus failing to give a comprehensive picture of the associations between disabilities and health in the general population. In this study, we aimed to provide a detailed health profile of children with different types of disabilities, which were carefully defined according to the classification of various authorities and researchers, and to explore the disability-specific associations with various health-related variables, including HRQoL, sleep-related QoL, physical functioning, emotional functioning, and social functioning among a large nonclinical sample of school-aged children in Hong Kong.

## 2. Materials and Methods

### 2.1. Study Design and Sample

During 2016 and 2017, we conducted a cross-sectional study on children attending primary or secondary schools (6–18-year-olds) in Hong Kong. This study included children with more severe disabilities attending special schools, children with less severe disabilities who were placed in ordinary schools for inclusive education, and children without any disability attending ordinary schools. For children with disabilities, children younger than 9 years of age, or children with any problem that made them incapable of completing the questionnaire alone, one of their parents or the major caregivers were asked to provide proxy reports. On the other hand, older children (10-year-olds or older) without any disability were asked to give self-reports.

### 2.2. Data Collection

We sampled ordinary primary and special schools with the sampling frames being sorted by the geographical district and the financing mode of the schools. A total of 78 schools were sampled and recruited, and 67 agreed to participate in the study (response rate of schools = 85.9%). All children with disabilities attending the sampled ordinary and special schools were invited to join the study, whereas children without disabilities were selected by a random sampling procedure. Children’s self-reports were completed with trained research assistants in a private and quiet room of the participating schools. Proxy reports from parents or major caregivers were conducted in the form of a questionnaire that was to be returned to researchers upon completion.

Ethics approval for the study was obtained from the Institutional Review Board of the University of Hong Kong and the Hospital Authority, Hong Kong West Cluster (Reference number UW12-529), and all study design aspects and procedures strictly followed the safety protocol. For each participant in the study, one of the parents or legal guardians provided written informed consent and, where applicable, children gave assent. All participants, including children and their proxies, were provided a thorough explanation of the purpose of the proposed study and their rights to refuse participation, to terminate the interview, and to ignore any item presented either verbally or in printed form. Anonymity and confidentiality were assured.

### 2.3. Measures

The 10-item Barthel Index of Activities of Daily Living (The Barthel ADL Index) was used to measure the physical functioning of children in various ADL, such as feeding, self-bathing, dressing, walking, and ascending or descending stairs [13]. Each item was rated on a three-point Likert scale. The total score ranged from 0 (indicating total dependence on others in ADL) to 20 (indicating total independence).

Sleep-related QoL was assessed with five items, which were developed based on two existing scales, the Children’s Sleep Habit Questionnaire and the Hong Kong Children Sleep Questionnaire [14,15]. The first three items asked if the child had any problem with (a) getting to sleep, (b) staying asleep, and (c) waking up too early. The fourth item asked about the satisfaction level of the child’s sleep quality, and the last item asked whether the sleeping problem, if any, negatively affected the child’s daily activities (such as emotional functioning and school functioning). All items were rated on a five-point Likert scale. In this study, higher scores demonstrated better sleep-related QoL.

The Chinese version of the Pediatric Quality of Life Inventory Generic Core Scale (PedsQL) was used to measure HRQoL among children [16]. The 23-item PedsQL is a multidimensional scale that measures child health-related quality of life by assessing children’s problems related to physical, emotional, social, and school functioning. The items are rated on a five-point scale. To facilitate analysis, we converted the item scores reversely to “100”, “75”, “50”, “25”, and “0”, respectively, with higher scores indicating better HRQoL. All item scores were added to give the total score, while the mean scores of each subscale were used as the subscale scores.

We measured the problems and difficulties in emotional and social functioning among children using four subscales of the Strengths and Difficulties Questionnaire (SDQ) [17]. Each of the four subscales contained five items describing issues related to (a) conduct problems, (b) hyperactivity, (c) emotional problems, and (d) peer problems. All items were rated on a three-point Likert scale. The total score ranged from 0 to 40 in this study, with higher scores indicating more severe problems and difficulties. Children were classified into three groups, namely “normal” (with total score of 15 or below), “borderline” (with total score from 16 to 19), and “abnormal” (with total score of 20 or above), according to the total scores of the reports.

We captured the disabilities status using items asking whether the child had ever received diagnosis of a specific disorder or problem. By integrating the disability categories as defined by other researchers and authorities [4,12], disabilities were categorized as follows: (a) physical disabilities, including restrictions in body movement, visual impairment, hearing impairment, and speech and language impairment; (b) learning and developmental disabilities, including attention deficit/ hyperactivity disorder (ADHD) and other special learning difficulties; (c) intellectual disabilities, or mental retardation; (d) internalizing disorders or mental illness, such as depression, anxiety, post-traumatic disorder, and other emotional disorders; and (e) autism spectrum disorder.

Some basic demographic and family background information of the children and their parents were also recorded. These included (a) individual variables: children’s gender, age, dependence on mobility assistance, and chronic illness, if any; (b) family structure: number of siblings living in the same household, parental marital status, living arrangement for the child, and major caregivers of the child; (c) disabilities or chronic illness among other family members including siblings and parents; and (d) financial stress of the family, in particular whether the parents were unemployed and whether the family was receiving social security.

### 2.4. Statistical Analysis

We computed descriptive statistics for the demographic and family variables, disabilities status, and health-related variables, and made comparisons between children with and without disabilities with chi-square tests, *t*-tests, and one-way analysis of variance (ANOVA), as appropriate. To examine the associations between different types of disabilities and children’s performance in regard to ADL, sleep-related QoL, and HRQoL, we used a series of multivariate regression models. Children’s ADL, sleep-related QoL, and HRQoL were the dependent variables, and types of disabilities were the independent variables. The models were adjusted for child individual factors, family structure factors, family member’s disability/chronic illness, and financial stress variables. To examine the association between different types of disabilities and children’s behavioral problems and difficulties (assessed as normal by strengths and difficulties questionnaire-SDQ), we used a series of logistic regression analyses. The analyses were adjusted for child individual factors, family structure factors, family member’s disability/chronic illness, and financial stress variables. All statistical analyses were conducted using Statistical Package for the Social Sciences (SPSS 24.0). In this study, *p*-values < 0.05 were regarded as statistically significant.

## 3. Results

The final sample (*n* = 4114) comprised 2417 child reports (58.8%) and 1697 proxy reports (41.2%) on children in Hong Kong (response rate = 94.9%). Table 1 summarizes descriptive statistics and comparisons of the demographic profile of the children in this study. We found generally higher proportions of boys, older children, and only children among the group with disabilities. This group was also found to be more likely to live with and be taken care of by single parents and grandparents than their counterparts, who were more likely to live with and be taken care of by both parents.

Table 2 shows the percentages of different types of disabilities among children by the type of school attended. Among the children attending special schools, learning and developmental disabilities were the most prevalent type of disability (74.9%), followed by intellectual disabilities (44.1%), autism spectrum disorders (35.1%), and physical disabilities (30.1%). Internalizing disorders, mental illnesses, or mood disorders was the least prevalent group (2.2%). Among the children attending ordinary schools, learning and developmental disabilities (5.6%) and physical disabilities (1.4%) were the most common types of disabilities. Internalizing disorders, mental illnesses, or mood disorders (0.7%), autism spectrum disorders (0.7%), and intellectual disabilities (0.1%) were the least common types of disabilities.

The findings on the four health-related variables as measured by the scales are summarized in Table 3. Overall, children with disabilities (a) scored lower in the Barthel ADL Index, showing poorer physical functioning in terms of ADL; (b) scored lower in the PedsQL and all of the four subscales, indicating poorer health-related QoL; and (c) were more likely to show abnormal difficulties in emotional and social functioning, as assessed by the SDQ, than their counterparts. Nonetheless, we did not find significant difference in the sleep-related QoL between the two groups of children.

Table 4 shows the findings of the multivariate regression and logistic regression models. We revealed that (a) restriction in body movement, visual impairment, speech and language impairment, and intellectual disabilities were associated with poorer ADL; (b) speech and language impairment and ADHD were negatively associated with sleep-related QoL, whilst intellectual disabilities were positively related to it; (c) all types of disabilities but hearing impairment were negatively associated with HRQoL; (d) speech and language impairment, ADHD, internalizing disorder, mental illness, and mood disorder, as well as autism spectrum disorder were all associated with an increased odds of showing abnormal behavioral difficulties; and (e) hearing impairment was the only type of disability that was not associated with hampered health-related variables after controlling for individual factors and family structure.

## 4. Discussion

We found significantly poorer physical, emotional, and social functioning among children with disabilities. Consistent with previous research [5,6,7,8,9,10,11,18,19,20], findings of the regression and logistic regression models showed that physical disabilities (except for hearing impairment) and intellectual disabilities were particularly associated with hampered physical functioning, whereas speech and language impairment, ADHD, autism spectrum disorder, and internalizing and mental disorders were associated with poorer emotional and social functioning.

There was a significant association between speech and language impairment and hampered physical functioning. To our best knowledge, this finding is the first piece of evidence for such an association. With regard to the possible association between language impairments and limited communications as well as hampered social interactions [21], we believe that the greater dependence on others across various ADL among children with language impairments might arise from the difficulties among those children in communicating with non-caregivers. For example, young children, especially those who have not been well trained for communication, might not be able to express their feelings and wills effectively to others and might thus rely heavily on parents or caregivers who are more familiar with their daily routines for assistance in feeding and toileting. Yet, due to the limitations surrounding the nature of this study, we cannot provide any empirical evidence for the mechanism underlying such an association.

Our findings also revealed that children with language disabilities might demonstrate more behavioral problems in terms of emotional and social functioning. On one hand, the association might be a direct one, as children with language impairments might have problems in social interactions with peers and find it hard to express themselves and communicate with others. On the other hand, the association might be an indirect one, and there might be underlying factors affecting the emotional and social functioning among these children. For example, according to a previous 3-year longitudinal study on 13 children with developmental disabilities [22], the severity of aberrant behaviors, such as aggression and extreme tantrums, significantly increased with the level of communication impairments of these children. In this case, the aberrant behaviors shown by children with language impairments might not only increase the likelihood of their conduct and emotional problems but might also hinder their healthy social interactions with others and restrict their participation in school settings [23].

Another surprising finding was the positive association between language impairment and sleep-related QoL. In one of the very few studies available on sleep quality among children with language impairment [24], children with language impairment reported fewer sleep problems than the control group. Another study demonstrated that sleep disturbance tended to affect children with autism but not those with language impairment [18]. Our study provided differentiating evidence and might warrant further research on the relationship between sleep problems and speech and language impairments in the future.

Consistent with some past research [25,26], our findings demonstrated that children with ADHD achieved poorer sleep-related QoL. A previous review found that about 30% of children with ADHD were affected by sleep problems including insomnia, delayed sleep phase, and fractured sleep [27]. However, there is currently no conclusive evidence of the direction of the association between sleep problems and ADHD in literature. Whereas the symptoms of ADHD, such as trouble staying still and controlling one’s behaviors, may lead to the difficulty getting to sleep, sleep disturbance itself may also lead to increased restlessness and impulsiveness among ADHD children via overcompensation [28].

As predicted, almost all types of disabilities were associated with poorer HRQoL when compared with the control group. The only exception was hearing impairment. Contrary to the finding of a recent meta-analysis [29], children with hearing impairments did not show poorer HRQoL than the general population in our study. The non-significant association might be partly explained by the lack of correlation between the level of speech perception and the QoL among individuals with hearing problems, as revealed in another recent study [30]. When the level of speech reception is not an important aspect affecting one’s HRQoL, children with hearing impairments may achieve good HRQoL as long as they do not have problems communicating with others.

Several limitations existed in this study. Potential biases might appear in self-reports and proxy reports with regards to the children’s experiences, despite our attempts to ask parents or caregivers who were most familiar with the children to be respondents. Also, the cross-sectional design did not allow for an investigation of the causal relationships among variables. This was especially obvious when considering that some types of disabilities might be influenced by health in a reciprocal way. The relatively small number of participants in several subgroups in the study (e.g., children with hearing impairments and children with internalizing disorders) might affect the comparisons between groups. Moreover, this study did not examine co-morbidity of disabilities, which might influence the relationships between variables. To facilitate reliable comparisons and to address the limitations of this study, future studies may replicate the study by increasing the sample size, using multiple informants, and using a longitudinal study design.

## 5. Conclusions

This study is among the first to explore the associations between different carefully defined types of disabilities and health-related aspects in one single, large sample of school-aged children. This study provides a detailed health-related profile of children with different types of disabilities and demonstrates that children with disabilities were generally poorer in physical functioning, emotional functioning, social functioning, and school functioning. This study extends previous research by exploring the disability-specific associations with HRQoL and other health correlates, and it successfully provides reliable empirical support for several disability-specific associations with functioning and QoL among children. These specific associations provide insights for the development and allocation of resources for disability-specific intervention programs for disabled children with different needs.

## Figures and Tables

**Table 1:** Summary of the individual and family characteristics of the children sample.

		Percentage	
		Group of Children	
Characteristics	Children with disabilities (*n* = 1101)	Children without disabilities (*n* = 3013)	Difference ^a^ (*p*-value)
Individual profile			<0.001
Gender			
Male	62.9%	50.3%	
Female	37.1%	49.7%	
Age (years), mean (Standard Deviation SD)	12.59 (3.97)	12.01 (3.40)	<0.001
Age group			<0.001
6–9 years	21.0%	18.7%	
9–12 years	20.6%	28.0%	
12–14 years	14.4%	14.8%	
14–18 years	31.0%	35.8%	
18 years or older	13.0%	2.8%	
Dependence on mobility assistance			<0.001
Wheelchair or other walking aids	5.4%	0	
Prosthesis	15.5%	0	
Other person’s aid when going upstairs/downstairs	2.7%	0	
None	76.3%	100%	
Chronic illness			<0.001
Hypertension	1.3%	1.2%	
Heart disease	15.0%	5.9%	
Asthma	18.1%	40.0%	
Diabetes	1.8%	0.6%	
Renal disease	2.7%	1.8%	
Cataract	3.5%	0	
Tuberculosis	0	0	
Peptic ulcer disease	0.4%	0.6%	
Skin disease	13.7%	24.1%	
Other(s)	36.7%	15.9%	
Family structure			
No. of siblings within the household			<0.001
None (i.e., only child)	44.9%	33.8%	
One	41.2%	47.7%	
Two or more	13.9%	18.5%	
Parents’ marital status			0.17
Married, or cohabiting	87.1%	89.1%	
Single, or widowed	10.7%	8.8%	
Missing	2.2%	2.1%	
Living arrangement for the child			<0.001
With both parents	73.2%	78.4%	
With single parent and grandparent(s)	3.7%	3.2%	
With single parent only	18.8%	13.0%	
Attending boarding school	4.6%	0.2%	
With other relatives	3.8%	2.8%	
Major caregiver(s) of the child			<0.001
Both parents	41.3%	54.9%	
Single parent and grandparent(s)	4.2%	4.4%	
Single parent only	39.0%	26.0%	
Staff at boarding school	4.8%	0.1%	
Other relatives	9.8%	7.1%	
Disability or chronic illness among family members			
Sibling with disability	11.8%	2.4%	<0.001
Sibling with chronic disease	5.6%	3.4%	<0.001
Father with disability	5.3%	1.2%	<0.001
Father with chronic disease	5.8%	1.5%	<0.001
Mother with disability	12.3%	10.0%	<0.001
Mother with chronic disease	9.4%	6.1%	<0.001
Financial stress of the family			
Father unemployed	5.6%	4.1%	<0.001
Mother unemployed	5.4%	6.8%	<0.001
Receiving social security	17.4%	10.0%	<0.001

^a^ Tested by chi-square tests or *t*-tests.

**Table 2:** Distribution of children by type of disability.

	Frequency (Percentage)	
	Type of School	
Type of disabilities ^a^	Special ^b^ (*n* = 873)	Ordinary (*n* = 3241)
Disability type		
(I) Physical disabilities	263 (30.1%)	46 (1.4%)
Restriction in body movement	83 (9.5%)	2 (0.1%)
Visual impairment	57 (6.5%)	28 (0.9%)
Hearing impairment	26 (3.0%)	11 (0.3%)
Speech and language impairment	97 (11.1%)	5 (0.2%)
(II) Learning and developmental disabilities	654 (74.9%)	180 (5.6%)
Specific learning difficulties, including dyslexia	357 (40.9%)	44 (1.4%)
Attention deficit/ hyperactivity disorder (ADHD)	297 (34.0%)	136 (4.2%)
(III) Intellectual disabilities	385 (44.1%)	4 (0.1%)
(IV) Internalizing disorder, mental illness, or mood disorder	19 (2.2%)	24 (0.7%)
(V) Autism spectrum disorder	306 (35.1%)	24 (0.7%)
Without disability	0 (0%)	3013 (93.0%)

^a^ Types of disabilities were determined by integrating the classification by the Education Bureau, Hong Kong, the World Health Organization (WHO), Turner et al.‘s study (2011) [4], and Jones et al.’s study (2012) [12]. Children or their proxy respondent could report more than one type of disabilities. ^b^ Special school services are provided by the Education Bureau to students with severe special learning needs in Hong Kong after the Bureau’s assessment. Some students with less severe special learning needs might be placed in mainstream (ordinary) schools.

**Table 3:** Descriptive statistics as measured by the four health-related measures.

	Mean Score (SD)/ Percentage (%)		
	Group of Children		
Health measure	Children with disabilities (*n* = 1101)	Children without disabilities (*n* = 3013)	Difference ^a^ (*p*-value)
Barthel ADL Index	17.00 (4.89)	19.49 (2.22)	<0.001
Sleep QoL	4.14 (0.65)	4.17 (0.60)	0.27
PedsQL	1517.12 (419.48)	1893.14 (328.73)	<0.001
Physical functioning	73.74 (23.66)	87.51 (14.53)	<0.001
Emotional functioning	71.01 (19.85)	76.60 (20.81)	<0.001
Social functioning	53.56 (28.25)	84.77 (18.14)	<0.001
School functioning	60.87 (20.46)	77.33 (17.72)	<0.001
SDQ, total difficulties (%)			<0.001
Normal	39.0%	69.0%	
Borderline	19.3%	14.4%	
Abnormal	37.0%	13.8%	

^a^ Tested by chi-square tests or *t*-tests. Barthel ADL Index: The Barthel Index of Activities of Daily Living; Sleep QoL: The Sleep-related Quality of Life Scale; PedsQL: The Pediatric Quality of Life Inventory Generic Core Scale; SDQ: The Strengths and Difficulties Questionnaire.

**Table 4:** Standardized β coefficients, adjusted odds ratios, and model summaries of the regression models showing the associations between different types of disabilities and health measures among children (*n* = 4114).

Type of Disability	Standardized β			Adjusted Odds Ratio (aOR) (95%CI)
	Barthel ADL Index	Sleep QoL	PedsQL	SDQ (Abnormal)
(I) Physical disabilities				
Restriction in body movement	−0.37 ***	−0.04	−0.15 ***	0.85 (0.49–1.65)
Visual impairment	−0.60 **	−0.02	−0.05 **	1.18 (0.65–2.15)
Hearing impairment	−0.01	0.02	−0.02	0.70 (0.27–1.82)
Speech and language impairment	−0.22 ***	−0.11 ***	−0.13 ***	2.37 (1.31–4.32) **
(II) Learning and developmental disabilities				
Specific learning difficulties, including dyslexia	−0.05 *	0.01	−0.07 ***	1.07 (0.74–1.56)
Attention deficit/ hyperactivity disorder (ADHD)	0.01	−0.07 **	−0.11 ***	2.20 (1.57–3.07) ***
(III) Intellectual disabilities	−0.18 ***	0.06 **	−0.17 ***	1.03 (0.75–1.41)
(IV) Internalizing disorder, mental illness, or mood disorder	0.01	−0.03	−0.04 *	3.46 (1.45–8.27) **
(V) Autism spectrum disorder	0.01	−0.002	−0.18 ***	2.61 (1.96–3.48) ***
Model statistics				
Adjusted R^2^	0.37	0.05	0.26	0.10
*F*-change in R^2^/χ^2^	75.06	9.44	52.14	9.24
*p*-value	<0.001	<0.001	<0.001	0.32

Barthel ADL Index Barthel ADL Index = The Barthel Index of Activities of Daily Living. Sleep QoL = The Sleep-related Quality of Life Scale. PedsQL = The Pediatric Quality of Life Inventory Generic Core Scale. SDQ = The Strengths and Difficulties Questionnaire. All models were adjusted for child individual factors, family structures factors, family member’s disability/chronic illness, and financial stress variables. * *p* < 0.05; ** *p* < 0.01; *** *p* < 0.001.
